# Toll-Like Receptor 4 Mediates Acute Lung Injury Induced by High Mobility Group Box-1

**DOI:** 10.1371/journal.pone.0064375

**Published:** 2013-05-17

**Authors:** Yuxiao Deng, Zhongwei Yang, Yuan Gao, Huan Xu, Beijie Zheng, Min Jiang, Jin Xu, Zhengyu He, Xiangrui Wang

**Affiliations:** 1 Department of Anesthesiology, Ren Ji Hospital, School of Medicine, Shanghai Jiao Tong University, Shanghai, China; 2 Institutes of Brain Science and Key Laboratory of Medical Neurobiology, Shanghai, China; National Institute of Infectious Diseases, Japan

## Abstract

**Background:**

Acute lung injury (ALI) is considered to be the major cause of respiratory failure in critically ill patients. Clinical studies have found that in patients with sepsis and after hemorrhage, the elevated level of high mobility group box-1(HMGB-1) in their circulation is highly associated with ALI, but the underlying mechanism remains unclear. Extracellular HMGB-1 has cytokine-like properties and can bind to Toll-like Receptor-4 (TLR4), which was reported to play an important role in the pathogenesis of ALI. The aim of this study was to determine whether HMGB-1 directly contributes to ALI and whether TLR4 signaling pathway is involved in this process.

**Methods:**

Recombinant human HMGB-1 (rhHMGB-1) was used to induce ALI in male Sprague-Dawley rats. Lung specimens were collected 2 h after HMGB-1 treatment. The levels of TNF-α, IL-1β, TLR4 protein, and TLR4 mRNA in lungs as well as pathological changes of lung tissue were assessed. In cell studies, the alveolar macrophage cell line, NR8383, was collected 24 h after rhHMGB-1 treatment and the levels of TNF-α and IL-1β in cultured medium as well as TLR4 protein and mRNA levels in the cell were examined. TLR4-shRNA-lentivirus was used to inhibit TLR4 expression, and a neutralizing anti-HMGB1 antibody was used to neutralize rhHMGB-1 both *in vitro* and *in vivo*.

**Results:**

Features of lung injury and significant elevation of IL-1β and TNF-α levels were found in lungs of rhHMGB-1-treated animals. Cultured NR8383 cells were activated by rhHMGB-1 treatment and resulted in the release of IL-1β and TNF-α. TLR4 expression was greatly up-regulated by rhHMGB-1. Inhibition of TLR4 or neutralization of HMGB1 with a specific antibody also attenuated the inflammatory response induced by HMGB-1 both *in vivo* and *in vitro*.

**Conclusion:**

HMGB-1 can activate alveolar macrophages to produce proinflammatory cytokines and induce ALI through a mechanism that relies on TLR-4.

## Introduction

Acute lung injury (ALI) is considered to be the major cause of rapid-onset respiratory failure in critically ill patients. Indeed, its severe form, acute respiratory distress syndrome, often results in multi-organ failure with a mortality of approximately 30–50% [1]. Recently, nonspecific inflammation has been found to play an essential role in the pathogenesis of this disorder [2], [3]. Our previous work [4], [5], [6], as well as the work of others, indicated that Toll-like receptor 4 (TLR4) mediates many forms of ALI, such as hemorrhagic shock-induced ALI and ventilator-induced ALI [7], [8]. Once TLR4 binds with its ligands, it further activates NF-κB through a MyD88-dependent pathway that ultimately stimulates the expression of proinflammatory cytokines, such as interleukin (IL)-1, IL-6, and tumor necrosis factor (TNF)-α leading to pathological changes in ALI [9], [10]. In addition, TLR4 can be activated by prototypical pathogen-associated molecular pattern (PAMP) and damage-associated molecular pattern (DAMP) proteins, such as HSP70 and High Mobility Group Box Protein-1 (HMGB-1) [11], [12].

HMGB1, as a nonhistone nuclear protein that acquires both intracellular and extracellular activities, was shown to bind with TLR4 [13], [14]. In the cell, it binds chromatin, interacting with both DNA and transcription factors, and thereby regulating transcription [15], [16]. Extracellular HMGB-1 has cytokine-like properties [13], [17] and has been implicated in the development of arthritis [18], activation of human monocytes[19], smooth muscle cell chemotaxis [20], and induction of adhesion molecules in endothelial cells [21]. More importantly, clinical data collected from critically ill patients showed that circulating levels of HMGB1 are increased in patients with infectious diseases and trauma [22], [23], [24]. In addition, in patients with sepsis and after hemorrhage, the elevated level of HMGB-1 in their circulation is highly correlated with the occurrence of ALI [24], [25]. However, the mechanisms underlying this strong correlation remain unclear.

Based upon these findings, we hypothesized that TLR4 can be activated by HMGB-1, which subsequently triggers the release of inflammatory cytokines and results in ALI. In this study, we show that HMGB-1 can stimulate the release of IL-1β and TNF-α both *in vitro* and *in vivo*. More interestingly, TLR4 expression was greatly up-regulated by recombinant human HMGB-1 (rhHMGB-1), and inhibition of TLR4 or administration of an HMGB-1 neutralizing antibody attenuated the HMGB-1-mediated inflammatory response.

## Materials and Methods

### Ethics statement

All animal work was approved by the Animal Care and Use Committee of the Shanghai Jiaotong University School of Medicine. All animals received humane care in accordance with the guidelines for animal care published by the United States' National Institutes of Health (NIH) for animal care (Guide for the Care and Use of Laboratory Animals, Department of Health and Human Services, NIH Publication No. 86–23, revised 1985).

### Experimental Animals

Male Sprague-Dawley rats (180–220 g) purchased from SLAC Laboratory Animal Company (Shanghai, China) were used in this study. Animals were housed under conditions of controlled temperature (22–24°C), 12-h light/dark cycles (8:00–20:00 light; 20:00–8:00 dark), and free access to food and tap water.

### Cell Culture

The alveolar macrophage cell line, NR8383, was obtained from the Cell Bank of the Chinese Academy of Sciences (Shanghai, China) and was maintained in a cell incubator (HF90, Healforce, Shanghai, China) at 37°C in 5% CO_2_-enriched air. The culture medium used was Ham's F-12 (Invitrogen, Carlsbad, CA, USA) media containing 15% fetal bovine serum (FBS; Hyclone).

### Inhibition of TLR4 expression

RNA interference (RNAi) was used to block translation of TLR4 mRNA. A specific RNAi lentivirus vector targeting TLR4 (shTLR4) and nontargeting shRNA control (shNT) were constructed and verified by the Shanghai GeneChem Co. Ltd. (Shanghai, China). The sense strand insert sequence was 5′ aaCCTAGAACATGTGGATCTT 3′. For animal studies, three weeks before HMGB-1 stimulation each rat was given 0.25 mL shTLR4 solution intratracheally, which contained 5×10^7^ TU. The shNT vector was used as a negative control. For the cell culture studies, NR8383 cells were seeded into six-well plates at a density of 2×10^5^ cells/well. Once the cells reached 80% confluence, they were transfected with either shTLR4 or shNT (2×10^5^ TU) complexed with complete medium and 5μg/ml polybrene according to the manufacturer's instructions. Fresh Ham's F-12 media containing 15% FBS was added 24 h post-transfection. The inhibition efficiency was assessed by western blot *in vivo* and *in vitro*.

### HMGB-1 stimulation

To generate the lung injury disease model, animals were instilled intratracheally with 50 μg or 100 μg of rhHMGB1 (Sigma-Aldrich, St.Louis, MO,USA) diluted in 0.25 ml of sterile phosphate-buffered saline (PBS). For cell culture experiments, NR8383 cells were treated with rhHMGB-1 at various concentrations (10, 50, or 100 ng/ml). Cells were collected 24 h after treatment for assessment in assays. According to the instructions for rhHMGB1 use, PBS was used as a control.

### Anti-HMGB-1 stimulation

To neutralize rhHMGB-1, animals were injected with 2 mg/kg of chicken neutralizing anti-HMGB1 polyclonal antibody (SHINO-TEST CORP, Kanagawa, Japan) via a tail vein 1 h before HMGB-1 stimulation according to the manufacturer's instructions. For cell culture experiments, NR8383 cells were treated with 50 μg/ml chicken anti-HMGB1 polyclonal antibody 1 h before HMGB-1 stimulation. The biological activity of the antibody was tested *in vivo*. A concentration of 50 μg/ml anti-HMGB-1 antibody was shown to completely block 100 ng/ml rhHMGB-1-mediated activities in NR8383 cells (data not shown). A chicken IgY antibody (Abcam, Cambridge, MA, U.S.A) was used as a control.

#### Histopathological Analysis

Animals were anesthetized with pentobarbital sodium (100 mg/kg) and the lungs were removed 2 h after HMGB-1 stimulation and fixed in 10% buffered formaldehyde solution overnight. The tissue was then embedded the next day. Serial slices of each sample from the apex to base were acquired and two random sections were selected. Sections 5 μm thick were stained with hematoxylin and eosin. Evaluations were performed by a pathologist who was blinded to the experimental groups using an Olympus BX51 microscope (Olympus, Japan). Ten fields were chosen randomly from each section (a total of 20 fields per rat) and examined at a magnification of 200×. A separate grade from 0 to 3 was calculated for each field depending on the number of interstitial infiltrated neutrophils or the maximum width of alveolar septa as an index of interstitial edema. The number of interstitial infiltrated neutrophils in each field was graded as follows: less than 5 = 0; 5-100 = 1; 100-200 = 2; more than 200 = 3. The maximum width of alveolar septa (actual size in μm) in each field was graded as follows: less than 5 = 0; 5-20 = 1; 20-50 = 2; more than 50 = 3. For each variable, a single score was calculated per rat by summing the field scores. Moreover, a total lung injury score was calculated as the sum of the two components.

### Enzyme Linked Immunosorbent Assay (ELISA)

Lungs were harvested 2 h after HMGB-1 administration and washed three times in PBS. The samples were then homogenized, centrifuged at 11,000 g at 4°C for 15 min, and the supernatant was collected. Protein was quantified with a BCA Protein Assay Kit (Thermo scientific, Rockford, IL, USA). The cell culture medium was collected 24 h after treatment. The levels of TNF-α and IL-1β in lungs and cultured medium were measured using commercial ELISA kits (R&D Systems, Minneapolis, MN, USA) and performed following the manufacturer's instructions.

### Western Blot Analysis

Protein extraction and concentration determination were performed using the same procedures as described for the ELISA. For western blot, 20 µg of total protein were run on 10% SDS-PAGE. Proteins were then electrotransferred to nitrocellulose filter membranes. The membranes were incubated in PBS containing 5% nonfat dry milk for 2 h at 25°C. The blots were then incubated for 2 h at 25°C with primary antibodies for TLR4 (1∶1000; Cell Signaling Technology, Danvers, MA, USA) and β-actin (1∶2000; Bioworld Technology, St. Louis Park, MN, USA) and then incubated with IRDye 800CW-conjugated goat anti-rabbit secondary antibody (1∶5000; Santa Cruz Biotechnologies, CA, USA) for 1 h at 25°C. The infrared fluorescence image was obtained using the Odyssey infrared imaging system (Li-Cor Bioscience, Lincoln, NE, USA), and the intensity of bands were quantified by Image-Pro Plus 5.1 software.

### Real-time quantitative PCR Analysis

Total RNA was extracted from lungs (n = 6 for each group) using the TRIzol method (Invitrogen, Carlsbad, CA, USA). Using 1 µg total RNA, the first strand of cDNA was synthesized with the AMV enzyme in a 20 µl reaction mixture (Takara). Then, 2 µl of the product were used for real-time quantitative PCR in a final volume of 20 µl using the gene-specific primers. The following primers designed with Primer Express Software were used: rat TLR4 5′CAGGGAGCACGAGGCTTCTAACC 3′ (sense) and 5′ CTTGTGCCCTGTGAGGTCGTTGA 3′ (antisense); rat GAPDH 5′ AGACCTCTATGCCAACACAGTGC 3′ (sense) and 5′ GAGCCACCAATCCACACAGAGT 3′ (antisense). The amplification conditions were as follows: 95°C, 30 s, 1 cycle; 95°C, 3 s and 62°C, 30 s for 40 cycles. The melting curve was then determined. Gene transcripts were quantified with SYBR Premix Ex Taq Kit (Takara). Data were calculated using the 2^-ΔΔCT^ method and presented as fold change of transcripts for the HMGB1 and TLR4 gene in the lungs of other groups compared to sham-operated rats (defined as 1.0-fold). Rat GAPDH was used as an internal control. The relative expression of the target gene was normalized to the level of GAPDH in the same cDNA preparation.

### Statistical Analysis

All values are expressed as means±standard deviation (SD). Analysis of variance (ANOVA) followed by Tukey's multiple comparison tests was used. A two-sided P<0.05 was considered statistically significant.

## Results

### HMGB-1-Induced ALI

**Figure 1:**
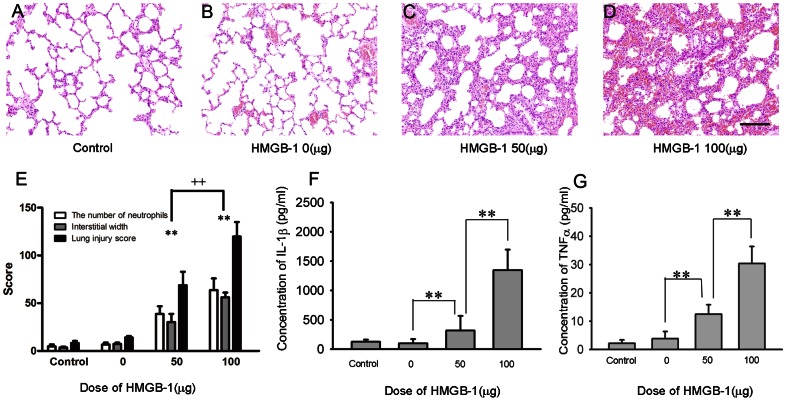
Lung inflammatory response after HMGB-1 treatment. Normal lung architecture was observed in the control group and 0 µg HMGB-1 group (A, B). Intense interstitial accumulation of neutrophils and edema was observed in 50 µg and 100 µg HMGB-1 groups (C, D). Rats exposed to 0 µg HMGB1 showed low lung histological scores. Intratracheal exposure of 50 µg and 100 µg of HMGB1 increased lung histological scores significantly (E). IL-1β and TNF-α levels in lungs were evaluated by ELISA. Compared with the control group, inflammatory mediators in the 0 µg HMGB-1 group were not significantly different, and marked increases were observed in 50 µg and 100 µg HMGB-1 groups (F, G). The increases in the 100 µg HMGB-1 group were higher than those in the 50 µg HMGB-1 group (F, G). Data are shown as the mean± SD, n = 6, *P<0.05, **P<0.01,scale bar 50 µm, original magnification 200×.

To examine whether HMGB-1 contributes to ALI, rats were instilled intratracheally with rhHMGB-1 at the indicated doses and lung histological observation was performed 24 h post-treatment. Samples in the control group in which animals were not treated showed a normal lung structure (Figure 1, panel A). In contrast, experimental groups displayed features of lung injury, including alveolar septal thickening, interstitial edema, vascular congestion, and neutrophil infiltration in the interstitium (Figure 1, panels C and D). In addition, intense interstitial accumulation of neutrophils and edema was observed, which indicated severe lung injury in groups exposed to 100 µg rhHMGB-1. Lung histopathological scores showed that change in histology in response to different doses of HMGB-1 treatment corresponded to the dose used (Lung injury score, 50 µg HMGB-1, 68.83±14.13 vs 8.33±2.16; 100 µg HMGB-1, 119.83±15.24 vs 8.33±2.16, vs control, **P<0.01, 50 µg HMGB-1 vs 100 µg HMGB-1, ++ *P*<0.01, n = 6, Figure 1, panel E).

We also determined the IL-1β and TNF-α levels in lungs 24 h after HMGB-1 treatment by ELISA. Compared with the control group, the IL-1β levels in the 0 µg HMGB-1 group had no significant change compared to control. In contrast, significant increases of IL-1β levels were found in the 50 µg and 100 µg HMGB-1 treated groups. IL-1β levels in the 100 µg HMGB-1 treated group was much higher than those in the 50 µg treated HMGB-1 group (control, 127.10±33.83 pg/ml; 0 µg HMGB-1, 146.83±21.50 pg/ml; 50 µg HMGB-1, 477.45±84.32 pg/ml; 100 µg HMGB-1, 1346.03±351.34 pg/ml; **P<0.01, n = 6; Figure 1, panel F). The TNF-α levels showed similar changes (control, 2.17±1.22 pg/ml; 0 µg HMGB-1, 3.50±3.71 pg/ml; 50 µg HMGB-1, 12.48±3.33 pg/ml; 100 µg HMGB-1, 30.35±6.02 pg/ml; **P<0.01, n = 6, Figure 1, panel G). These data suggest that HMGB-1 treatment triggers severe ALI.

### Extracellular HMGB-1 induces the production of proinflammatory cytokines in NR8383 cells

**Figure 2:**
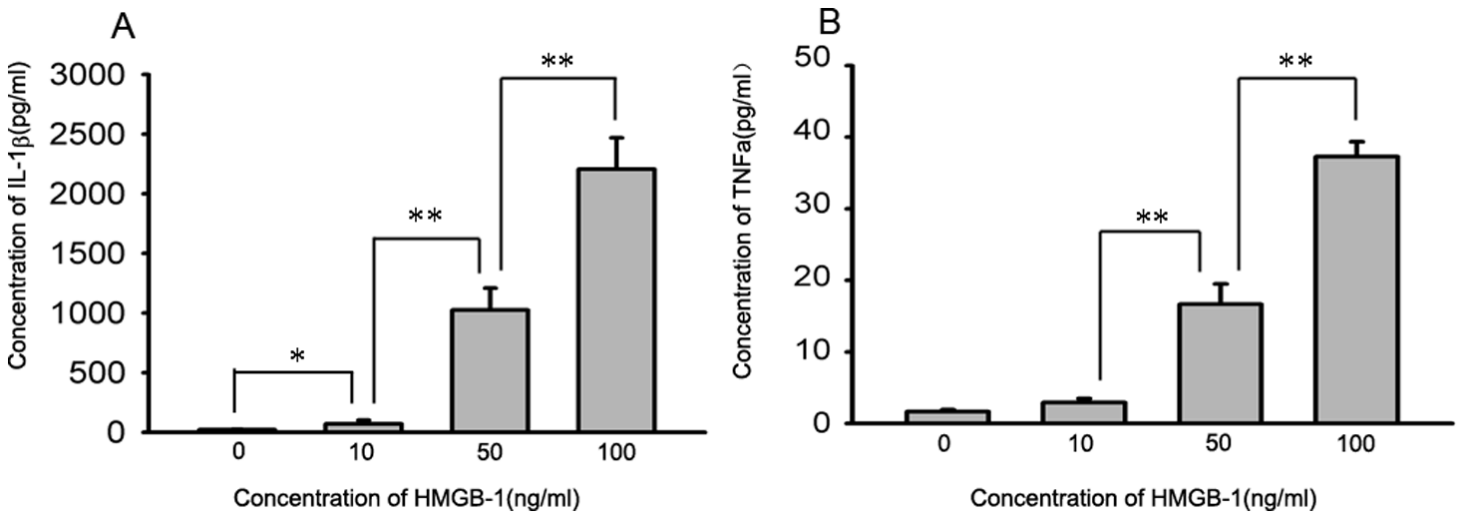
IL-1β and TNF-α levels in culture media change after HMGB-1 stimulation in NR8383. NR8383 cells were stimulated with rhHMGB-1at different concentrations and then the IL-1β and TNF-α levels in the culture media were evaluated by ELISA assay at 24 h post-treatment. After treatment, the IL-1β levels increased slightly in the 10 ng/ml HMGB-1 group. In the 50 and 100 ng/ml HMGB-1 groups, the levels of IL-1β increased dramatically (A). Compared with the control group, TNF-α levels did not change in the 10 ng/ml HMGB-1 group, but clearly increased in the 50 and 100 ng/ml HMGB-1 groups (B). Data are shown as the mean±SD, n = 6, *P<0.05, **P<0.01.

To test the effect of HMGB-1 *in vitro*, macrophages were treated with rhHMGB-1 and then the levels of IL-1β and TNF-α in the culture supernatants were determined by ELISA 24 h post-treatment. Compared with the control group, IL-1β levels in the 10 ng/ml HMGB-1 treated group were slightly increased. As the dose of HMGB-1 treatment increased, significant elevation of IL-1β levels were found in the 50 ng/ml and 100 ng/ml HMGB-1 treated groups(control, 22.35±2.5; 10 ng/ml HMGB-1, 70.8±31.77; 50 ng/ml HMGB-1, 1026.1±182.13; 100 ng/ml HMGB-1, 2205.95±262.77; *P<0.05, **P<0.01, n = 6; Figure 2, panel A). The TNF-α levels did not change in the 10 ng/ml HMGB-1 treated group, but were clearly elevated in the 50 and 100 ng/ml HMGB-1 treated groups (control, 1.64±0.30; 10 ng/ml HMGB-1, 2.90±0.58; 50 ng/ml HMGB-1, 16.67±2.81; 100 ng/ml HMGB-1, 37.28±2.10; **P<0.01, n = 6; Figure 2, panel B). These data demonstrate that exogenous HMGB-1 can induce the production of proinflammatory cytokines in macrophages.

### HMGB-1 upgrades TLR4 expression *in vitro* and *in vivo*

**Figure 3:**
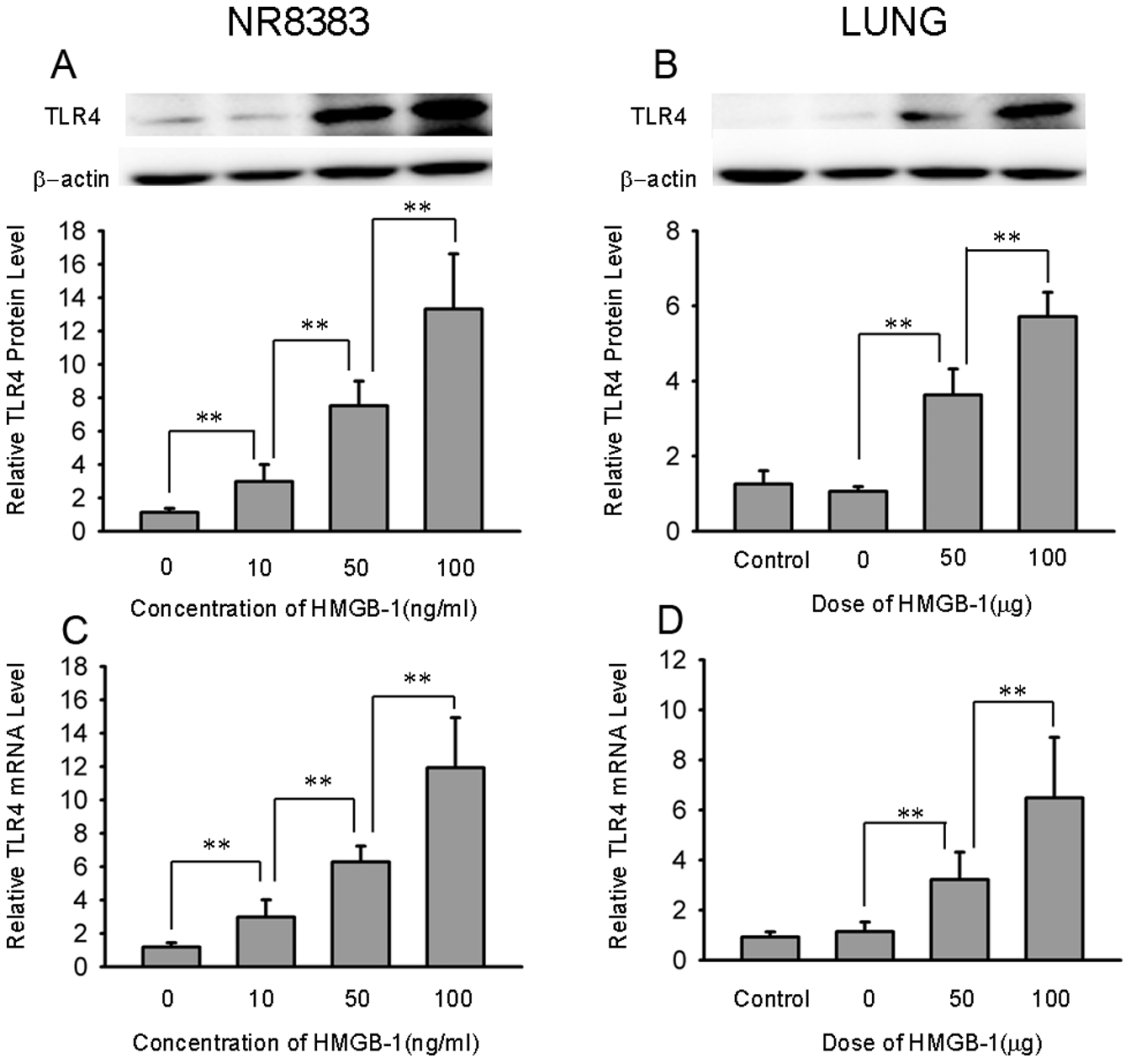
Expression of TLR4 after HMGB-1 treatment *in vitro* and *in vivo*. The expression of TLR4 was assessed by western blot analysis as well as real-time quantitative PCR analysis. For the *in vitro* studies, the expression of TLR4 protein and TLR4 mRNA levels were elevated after HMGB-1 stimulation, and every group displayed significant elevation (A, C). For the *in vivo* studies, the TLR4 protein and TLR4 mRNA levels in the 0 µg HMGB-1 treated group were comparatively equal to that of control. In addition, after HMGB-1 stimulation, the protein and mRNA levels were both clearly increased in a dose-dependent manner (B, D). Data are shown as the mean±SD, n = 6, **P<0.01.

To investigate the molecular mechanisms of HMGB-1-induced lung injury, the expression of TLR4 protein was assessed by western blot analysis and expression of TLR4 mRNA was assessed by real-time quantitative PCR analysis. In the *in vitro* studies, the expression of TLR4 protein gradually increased as the HMGB-1 concentration increased as compared to baseline(**P<0.01; n = 6, Figure 3, panel A). Similarly, the expression of TLR4 mRNA of each group was significantly elevated after HMGB-1 stimulation (**P<0.01, n = 6; Figure 3, panel B). In the *in vivo* studies, TLR4 protein and mRNA levels in the 0 µg HMGB-1 treated group were relatively equal to that of the control. In addition, after HMGB-1 stimulation, the levels of TLR4 protein and mRNA significantly increased in a dose-dependent manner (**P<0.01, n = 6; Figure 3, panel C and D). These data showed direct evidence that HMGB-1 induces TLR4 expression.

### TLR4 plays a role in the coordination of HMGB-1-induced lung injury

**Figure 4:**
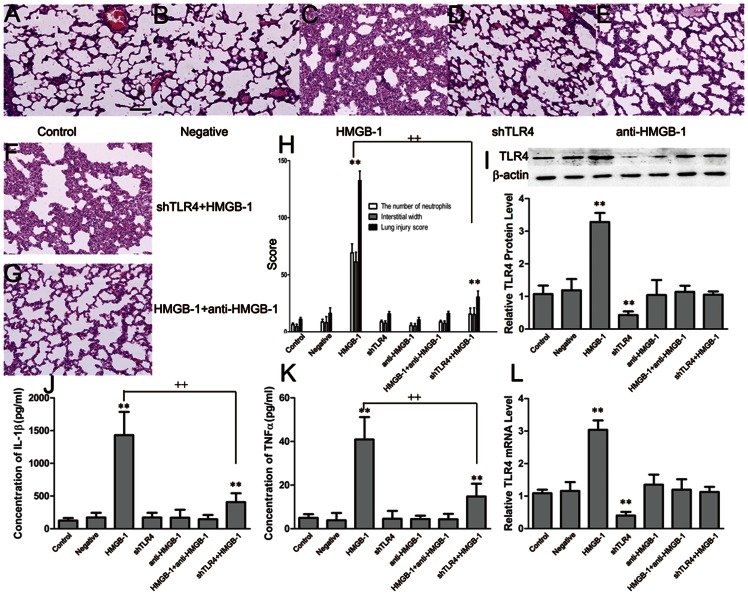
The effect of TLR4 inhibition on HMGB-1-induced ALI. RNAi was used to inhibit the expression of TLR4, and the animals received 100 µg of rhHMGB-1. Lung histological observation was performed after transfection and 24 h after HMGB-1 treatment. The negative and shTLR4 groups displayed normal lung structure similar to the control (A, B, and D) Stronger ALI effects were seen in the HMGB-1 group (C), but weaker ALI effects were observed in the shTLR4+HMGB-1 group (F).The anti-HMGB-1 and HMGB-1+anti-HMGB-1 groups also showed normal lung structure(E, G). Lung histopathological scores showed the degree of interstitial accumulation of neutrophils and edema: the HMGB-1 group was the highest, and the shTLR4+HMGB-1 was clearly lower than the shNT+HMGB-1 group, but still higher than the other three groups (H). The levels of TNF-α and IL-1β in lung were analyzed by ELISA after transfection and 24 h after HMGB-1 administration. The levels of IL-1β in lung were not significantly different between the control, negative, shTLR4, and anti-HMGB-1 groups. After treatment with HMGB-1, the levels of IL-1β and TNF-α increased dramatically in the HMGB-1 group and increased slightly in the shTLR4+HMGB-1 group, but did not change in the HMGB-1+anti-HMGB-1 group (J, K). TLR4 protein expression was measured by western blot analysis and TLR4 mRNA expression was measured by real-time quantitative PCR after transfection and 24 h after HMGB-1 administration. The TLR4 protein and mRNA levels were very low in the shTLR4 group, but dramatically increased in the HMGB-1 group. The levels of TLR4 expression in the other five groups were equal to each other (I, L). Data are shown as the mean±standard deviation (SD), n = 6, *P<0.05, **P<0.01 vs. control, ++ *P*<0.01 vs. HMGB-1.

To evaluate the potential role of TLR4 in HMGB-1-induced lung injury, we used RNAi to inhibit the expression of TLR4. As a control, animals were instilled intratracheally with 0.25 ml of sterile PBS only. For the HMGB-1, shTLR4+HMGB-1, and HMGB-1+anti-HMGB-1 groups, the animals were instilled intratracheally with 100 µg of rhHMGB1 diluted in 0.25 ml of sterile PBS. Lung histological assessment was performed after transfection and 24 h after HMGB-1 treatment. There were no pathological changes in control and negative control animals (Figure 4, panel A and B). The rats in the shTLR4 and anti-HMGB-1 groups displayed no evidence of neutrophils and edema in the pulmonary interstitium (Figure 4, panel D and E). Alveolar septal thickening, interstitial edema, vascular congestion, and neutrophil infiltration in the interstitium were found in the group stimulated by the addition of HMGB-1 (Figure 4, panel C). However, in the shTLR4+HMGB-1 group, we found a reduction in the severity of the lung injury, presumably due to the inhibition of TLR4 (Figure 4, panel F). Moreover, based on the neutralizing effect of the antibody, there were also no evidence of neutrophils and edema in the pulmonary interstitium in the HMGB-1+anti-HMGB-1 group (Figure 4, panel G). Lung histopathological scores showed neutrophil infiltration and interstitial edema after treatment of HMGB-1, and inhibition of TLR4 reduced these histological changes (Lung injury score, HMGB-1, 132.43±8.41 vs. 11.03±1.52; shTLR4+HMGB-1, 30.46±5.04 vs. 11.03±1.52, vs. control, **P<0.01, shTLR4+HMGB-1 vs. HMGB-1, ++ *P<*0.01, n = 6, Figure 4, panel H).

The tissue levels of TNF-α and IL-1β were analyzed by ELISA after transfection and 24 h after HMGB-1 treatment. There was no significant difference in the levels of IL-1β in lungs between the control, negative, shTLR4, and anti-HMGB-1 groups. After treatment with HMGB-1, the levels of IL-1β were dramatically elevated in the HMGB-1 group (1431.05±354.96 vs. 125.88±38.59 pg/ml vs. control, respectively; **P<0.01, n = 6, Figure 4, panel J) and slightly increased in the shTLR4+HMGB-1 group (407.73±135.16 vs. 125.88±38.59 pg/ml vs. control, respectively; **P<0.01; 407.73±135.16 vs. 1431.05±354.96 pg/ml vs. HMGB-1, respectively; ++*P<*0.01, n = 6, Figure 4, panel J). TNF-α levels in the lungs were equal in the control, negative, shTLR4, and anti-HMGB-1 groups. Similar to IL-1β levels, treatment with HMGB-1 caused a significant increase in TNF-α levels, but the increase was higher in the HMGB-1 group than that in the shTLR4+HMGB-1 group (control, 4.97±1.69 pg/ml; HMGB-1, 40.92±10.24 pg/ml; shTLR4+HMGB-1, 14.79±5.82 pg/ml, **P<0.01 vs. control, ++*P<*0.01 vs. HMGB-1, n = 6, Figure 4, panel K). The neutralizing antibody alleviated the significant increase in IL-1β and TNF-α levels caused by HMGB-1 (Figure 4, panel J and K).

The levels of TLR4 expression in lung tissue were measured by western blot analysis and real-time quantitative PCR after transfection and 24 h after HMGB-1 treatment. TLR4 expression was very low in the shTLR4 group, which was even lower than that of the control, confirming the inhibition of TLR4. Treatment with HMGB-1 resulted in a dramatic increase in TLR4 expression in the HMGB-1 group, but both TLR4 protein and mRNA levels in the shTLR4+HMGB-1 group had no significant change compared to those of the control. TLR4 expression in the HMGB-1+anti-HMGB-1 group was also equal to the control group, which confirmed the effect of the neutralizing antibody. (**P<0.01 vs. control, n = 6, Figure 4, panel I and L).

#### TLR4 inhibition diminishes the inflammatory response induced by HMGB-1 *in vitro*

**Figure 5:**
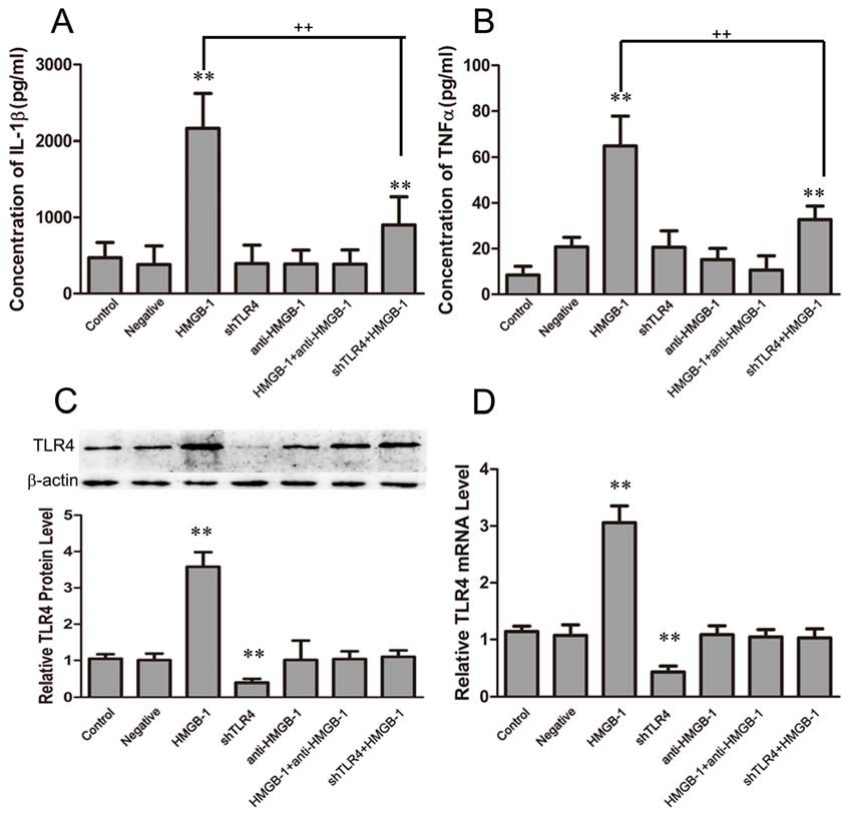
The effect of TLR4 inhibition on the inflammatory response by HMGB-1-treated NR8383 cells. RNAi was used to inhibit TLR4 expression in NR8383 cells. The levels of IL-1β and TNF-α in the culture supernatants were determined by ELISA after transfection and 24 h after HMGB-1 administration. The levels of IL-1β and TNF-α in negative, shTLR4, and anti-HMGB-1 groups were comparable to the control group. After treatment with HMGB-1, the IL-1β and TNF-α concentrations in the HMGB-1 group were higher than those in the shTLR4+HMGB-1 group, while there were no changes in these concentrations in the HMGB-1+anti-HMGB-1 group (A, B). The expression of TLR4 was assessed by western lot and real-time quantitative PCR after transfection and 24 h after HMGB-1 administration. After transfection, The TLR4 protein and mRNA levels were very low in the shTLR4 group, decreasing by approximately 60%. After treatment with HMGB-1, the levels of TLR4 protein and mRNA in the shTLR4+HMGB-1 group returned to the levels of the control. In addition, the levels of TLR4 protein and mRNA remained the same in the anti-HMGB-1 and HMGB-1+anti-HMGB-1 groups (C, D). Data are shown as the mean±SD, n = 6, **P<0.01 vs. control, ++ *P*<0.01 vs. shNT+HMGB-1.

To observe whether inhibition of TLR4 can diminish the inflammatory response induced by HMGB-1 *in vitro*, we used RNAi to inhibit TLR4 expression in NR8383 cells. The concentration of rhHMGB-1 used to activate NR8383 was 100 ng/ml. The levels of IL-1β and TNF-α in the culture supernatants were determined by ELISA after transfection and 24 h after HMGB-1 administration. We found that the levels of IL-1β and TNF-α in the negative and shTLR4 groups remained comparatively equal to that of the control, after treatment with HMGB-1, the concentration of IL-1β and TNF-α both elevated dramatically, but the degree of this increase in the two factors was different. In the HMGB-1 group, the concentrations of IL-1β and TNF-α increased by almost 4-5-fold compared to baseline levels, while the concentrations increased slightly in the shTLR4+HMGB-1 group (concentration of IL-1β, control, 470.93±201.53; HMGB-1, 2163.85±458.19; shTLR4+HMGB-1, 900.00±370.31; concentration of TNF-α, control, 8.45±3.83; HMGB-1, 64.93±12.89; shTLR4+HMGB-1, 32.71±5.88, **P<0.01 vs. control, ++*P<*0.01 vs. HMGB-1,n = 6, Figure 5, panel A and B). These data suggest that the inhibition of TLR4 may attenuate the production of proinflammatory cytokines in macrophages after HMGB-1 treatment. The use of a neutralizing antibody also alleviated the significant increase in IL-1β and TNF-α levels caused by HMGB-1 (Figure 5, panel A and B).

The expression of TLR4 was also assessed by western blot analysis as well as by real-time quantitative PCR analysis. After transfection, the levels of TLR4 protein and mRNA in the negative group were similar to control, but the level increased dramatically in the HMGB-1 group due to HMGB-1 treatment. After inhibition of TLR4, the level of TLR4 protein and mRNA decreased approximately 60% compared to control. After treatment with HMGB-1, the level of TLR4 protein and mRNA also increased, but the increase was still less than that of the control (**P<0.01 vs. control, ++*P<*0.01 vs. shNT+HMGB-1, n = 6, Figure 5, panel C and D). These data indicate that TLR4 inhibition reduced the downregulation of the inflammatory response induced by HMGB-1 *in vitro*. The effect of the neutralizing antibody was also confirmed in *in vitro* studies, as both the protein and mRNA levels of TLR4 in the HMGB-1+anti-HMGB-1 group were not significantly different from those of the control (Figure 5, panel C and D).

## Discussion

Based on clinical studies, recent data have shown that HMGB-1 concentrations in the circulation are elevated in patients with sepsis and trauma, and that this increase correlates with the development of ALI [24], [25]. Therefore, we hypothesize that HMGB-1 may play an essential role in the pathogenesis of ALI.

To determine whether HMGB-1 might induce ALI in rats, we instilled rats intratracheally with rhHMGB-1 and then observed the lung histology 24 h after treatment. We found interstitial accumulation of neutrophils and edema, which accounted for lung injury. At the same time, the levels of IL-1β and TNF-α in the lung were significantly elevated after treatment with HMGB-1. These results are similar to previous observations in mice, which showed that a prominent acute lung inflammatory injury was elicited with HMGB1 instilled intratracheally [24], [25], [26]. A widely used model of ALI is the intratracheal administration of endotoxin [27]. In this model, major pathological changes include a pulmonary inflammatory response with neutrophil infiltration and early increases in proinflammatory cytokines, such as IL-1β and TNF-α. Taken together with data from our experiments, we can conclude that ALI may be induced by HMGB-1 in a similar manner as lipopolysaccharide (LPS). To confirm the correlation between HMGB-1 and ALI, we instilled rats intratracheally with rhHMGB-1 at different doses and found that the lung histopathological scores and levels of IL-1β and TNF-α in the lung clearly increased in a dose-dependent manner. Therefore, our findings demonstrate that HMGB-1 alone induces ALI in a dose-dependent manner.

We also found that HMGB-1-induced ALI was mitigated by administration of HMGB-1 neutralizing antibody, which excluded the hybrid effect of ALI induced by nonspecific factors, such as contamination of endotoxin in rhHMGB-1. In another experiment with respect to cardiac pulmonary bypass (CPB)-induced ALI, anomalous accumulation of HMGB-1 was detected in lung tissue at different times after CPB (data not shown), implying that HMGB-1 may be involved in the mechanism of CPB-induced ALI. However, due to technical difficulties in establishing CPB in mice, a previous HMGB-1-induced ALI mouse model could not be applied in this study of the mechanism of CPB-induced ALI mediated by HMGB-1. Thus, our present study based on the rat model of HMGB-1-induced ALI provided a foundation for further investigation of the mechanism of CPB-induced ALI mediated by HMGB-1.

Alveolar macrophages have unique characteristics, as the alveoli serve as the interface between the outside and inside of the body in the respiratory system. These cells function by providing a defense against foreign microorganisms invading through the respiratory system. During the innate immune response, the overactivation of alveolar macrophages is one of the most critical events for the development of ALI [28]. Based on these findings, we chose the rat alveolar macrophage cell line, NR8383, as the model cell line for *in vitro* experiments. We found that levels of IL-1β and TNF-α significantly increased after HMGB-1 treatment. Morever, the increase in IL-1β and TNF-α levels correlated with the increase in the HMGB-1 concentration in the culture medium,which was reversed after administration of an HMGB-1 neutralizing antibody. Therefore, our findings provide evidence that alveolar macrophages are activated by HMGB-1, which results in the release of inflammatory mediators. Together with our *in vitro* experimental data, these findings could help illustrate mechanisms underlying the correlation between elevated levels of HMGB-1 in patients with trauma and hemorrhage and the occurrence of ALI. HMGB-1 is an important extracellular mediator in both local and systemic inflammation. There is also accumulating evidence that anti-inflammatory cytokines interleukin-(IL-)10 could alleviate an acute lung inflammatory response by inhibiting inappropriate secretion of HMGB-1 from lung tissue [29]. Thus, therapeutic treatment that specifically neutralizes overexpressed HMGB-1 may be a promising strategy in future clinical therapy for ALI.

TLR4 has been well documented as a pattern recognition receptor in acute infection-induced lung injury [30], [31]. Recent studies suggest that TLR4 signaling is actively involved in the pathogenesis of noninfectious lung injury [32], [33]. Although previous studies have demonstrated that ALI can be induced by HMGB-1 [24], [25], [34] and proinflammatory cytokines, such as TNF-α, IL-1β, IL-6, IL-8, and MIP-2β, which are produced by macrophages activated with HMGB-1 [14], [35], the underlying mechanism has not been fully elucidated. Our present study shows direct evidence that TLR4 may play a key role in HMGB1-mediated ALI. In animal studies, proinflammatory cytokines as well as TLR4 protein and mRNA increased significantly after HMGB-1 treatment, and the lung tissue had clear pathological changes. In addition, after TLR4 inhibition, rats treated with HMGB1 exhibited reduced lung injury as well as levels of proinflammatory cytokines in the lung tissue. The genetic reduction of TLR4 expression *in vitro* ameliorated HMGB-1-induced inflammation responses compared to nontargeting shRNA control. Since pulmonary macrophages are one of the most crucial inflammatory cytokine-producing cells, our study indicated that the activation of pulmonary macrophages mediated by TLR4 plays an important role in HMGB-1-induced ALI.

There were also some limitations in the present study. For example, we found that after TLR4 inhibition, treatment with HMGB-1 could still induce mild damage and activate macrophages in lung tissue both *in vitro* and *in vivo*. This could be a result of the RNAi only inhibiting 60% of the total TLR4 expression, and could also indicate that there are other pathways activated. Besides TLR4, HMGB-1 has been found to interact with other receptors, such as toll-like receptor 2 (TLR-2) and the receptor for advanced glycation end products (RAGE). As a transmembrane protein, RAGE binds to HMGB-1 and leads to activation of NF-κB, ERK1/2, p38, and SAPK/JNK kinases [20], [27], [28], [35], [36], [37]. However, RAGE only plays a minor role in HMGB-1-mediated macrophage activation [26]. HMGB-1 could rapidly interact with TLR-2 on RAW macrophage-like cells [38], and inhibition of TLR-2 expression in these cells by transfection with a dominant negative construct abolished HMGB-1-induced NF-κB activation [39]. The roles of TLR2 and RAGE in HMGB-1induced ALI need to be explored in future studies. However, for this study, our data support the idea that TLR4 is the major pathway that mediates the HMGB-1-induced ALI.

## Conclusion

HMGB-1 can induce alveolar macrophages to produce proinflammatory cytokines and induce ALI through a mechanism that relies on TLR-4.
